# Prevalence of Eating Disorders among Competitive Rowers

**DOI:** 10.3390/sports12100264

**Published:** 2024-09-24

**Authors:** Viola Keczeli, Andrea Gubicskóné Kisbenedek, Zsófia Verzár, Anita Hulman, Iván Petrov, Ferenc Ihász, Zoltán Alföldi

**Affiliations:** 1 Doctoral School of Health Sciences, Faculty of Health Sciences, University of Pécs, 7621 Pécs, Hungary; 2 Institute of Nutritional Sciences and Dietetics, Faculty of Health Sciences, University of Pécs, 7621 Pécs, Hungary; 3 Institute of Sports Sciences, Faculty of Psychology and Pedagogy, Eötvös Lóránd University, 9700 Szombathely, Hungary; 4 Institute of Physiotherapy and Sports Science, Faculty of Health Sciences, University of Pécs, 9700 Szombathely, Hungary

**Keywords:** eating disorder, rowing, weight-related sport, lightweight, openweight

## Abstract

Internationally, few studies have been written on the prevalence of eating disorders among rowers, and there is no research on the subject in Hungary. This is particularly important in weight-related sports such as rowing. The aim of this study is to assess the prevalence of eating disorders among rowers. A quantitative cross-sectional study was conducted in the summer of 2023. In a non-random convenience sampling, our target population consisted of certified competitive rowers (*n* = 222). In addition to socio-demographic, performance-related questions, the anonymous, self-completed questionnaire used the validated The Eating Disorder Inventory (EDI). Results were considered significant when *p* < 0.05. A total of 57 lightweight (25.67%) and 165 openweight (74.33%) rowers participated in the study. On the perfectionism subscale and the interoceptive awareness subscale, rowers competing in the lightweight category scored significantly higher (*p* = 0.03; *p* = 0.05). Scores on the EDI subscales, gender and BMI data did not show significant relationships. Overall, rowers in the openweight group scored higher on the bulimia subscale, but no rowers who met all criteria and could be identified as having eating disorders. Rowers who have only competed in national championships and not in international competitions were more likely to reduce stress levels by eating. The study showed that the prevalence of eating disorders among rowers is no higher than in the general population. However, continued assessment, further extensive research and education of rowers is essential in this area, as weight-related sports will always be a risk group for eating disorders.

## 1. Introduction

As defined by the American Psychiatric Association in 2019, the eating disorder is a psychosomatic illness in which the individual’s food intake is severely disturbed, resulting in a change in eating habits and associated thoughts and feelings. A patient becomes obsessed with food, body weight and body image [1]. There are three main types of eating disorders: anorexia nervosa, bulimia nervosa and binge eating disorder. Eating Disorders (EDs) are complex diseases, with biological factors involved in the predisposition, development and maintenance of the disorder, psychological and social factors, such as personality traits, family history of eating disorders, and individual and family stressors [2,3,4]. Although traditionally seen as a sport for elite schools and colleges, rowing is a founding Olympic event, and the popularity of rowing is growing every year by people of all ages and abilities. Rowing should not only be an accessible sport for elite athletes, but also an excellent hobby for health and recreation. At both the collegiate and Olympic levels, male and female rowers have two weight categories: openweight (no weight requirements) and lightweight. Men: crew average 70 kg (154.3 lb/11 st 0.3 lb)—no rower over 72.5 kg (159.8 lb/11 st 5.8 lb). Women: crew average 57 kg (125.6 lb/8 st 13 lb)—no rower over 59 kg (130.0 lb/9 st 4 lb). Because of these two weight categories, especially the lightweight, we can say that rowing is a weight-related sport. The prevalence of eating disorders varies widely depending on the population examined, high rates have been documented in female athletes in previous research, [5,6] particularly those involved in weight-related sports. Among adolescent and young adult male athletes, research concerning eating disorders is limited compared with female counterparts, but increasing evidence indicates that they may be at unique risk for unhealthy exercise and eating behavior. Therefore, in this study we focused on both sexes to assess the prevalence of eating disorders in the sample of rowers. Sports with previously documented weight-related eating disorders include mainly distance running, as well as aesthetic sports such as figure skating, gymnastics, and ballet, in which the athlete’s appearance and airy, effortless existence are very important [7,8,9,10]. Weight-related sports also include weight-dependent sports, which require athletes to adapt to very strict weight classes on the day of competition, such as wrestling, boxing and lightweight rowing. Perfectionism plays a role in the psychological impact of disordered eating in an athlete, acting both as a symptom and a risk factor for disordered eating. To maximise strength, power, and endurance, lightweight rowers are often naturally heavier than the maximum weight and use acute weight-loss methods, primarily hypohydration and nutrient restriction, before competition [11,12]. In 2016, Joy and colleagues conducted a study of 522 (*n* = 522) elite female athletes and 448 (*n* = 448) non-athletes, using questionnaires, clinical examinations and interviews. Joy determined that 13–18% of athletes were diagnosed with an eating disorder, compared to only 5% of the non-athlete control group [13]. In a similar but large study involving 1620 athletes and 1696 controls, similar results were found: 20% of female athletes met the criteria for an eating disorder, compared to 9.5% of the control group [14]. These research findings highlight the importance and inescapability of this problem in the athlete community. Managing eating disorders may require not only the help and support of a competent professional, but also the help and support of relatives or friends. Not only can the performance of athletes deteriorate, but in the long term their mental and physical health can be seriously affected. The sooner athletes with risk factors are identified and eating disorders are treated, the better the chances of recovery. Without treatment, the disease can lead to significant physical and psychosocial consequences that can lead to high mortality rates due to anorexia [15,16]. Once an eating disorder is diagnosed, a highly trained and experienced multidisciplinary health team should care for the athlete, providing personalised, patient-centred treatment or partial suspension from competition. The aim of this study was to assess the proportion of competitors at the 2023 Hungarian Rowing Championships with identifiable eating disorders and what factors might potentially influence the development of eating disorders among athletes. Additionally, we aimed to explore whether there is a relationship between athletes’ performance and the prevalence of eating disorders.

## 2. Materials and Methods

### 2.1. Participants

For this non-representative research, 222 (*n* = 222) rowers volunteered to participate in the survey using a non-random, convenience sampling. Non-random sampling is a major limitation in this research, as it can lead to selection bias. It can negatively affect the generalizability of results. The respondents are all competitors in the 2023 Hungarian Rowing Championships, registered athletes with a sports medical licence, certified by 21 sports clubs. The championship was held in Szeged, Hungary, from 6 to 9 July 2023. Competitors under the age of 18 had to obtain parental consent to participate in the research.

### 2.2. Measures

#### 2.2.1. The Eating Disorder Inventory

The Eating Disorder Inventory (EDI) was developed by Garner’s research group (1983). The tests used in earlier years were designed to assess behavioral symptoms of eating disorders, but did not assess associated psychopathological features. To fill these gaps, the EDI was developed, based on a multidimensional concept of eating disorders and assessing different aspects of the emotional and affective factors that best characterize them [17]. The multidimensional concept of eating disorders is now mostly used in various epidemiological screening and clinical status measures. Since then, several versions have been developed and translated into several languages. The Hungarian version of EDI has been validated by Ferenc Túry and his colleagues in 1997 [18].

The EDI is a multiple-choice self-report questionnaire using a Likert-type scale with 64 items, assessing different aspects of cognitive and emotional factors specific to eating disorders in 8 subscales: urge to be thin, bulimia, body dissatisfaction, sense of inadequacy, perfectionism, interpersonal distrust, interoceptive awareness, fear of growing up. The first 3 subscales measure attitudes and/or behaviors related to eating and body shape, while the other 5 factors assess personality traits identified as core psychopathological features of anorexia nervosa (AN). The characterisation of each subscale is given below.

1. The urge to be thin: This subscale includes items reflecting excessive preoccupation with dieting, worrying about weight, striving to lose weight, being thin, intense fear of gaining weight.
2. Bulimia: Items indicate tendencies to overeat uncontrollably and may be followed by a strong urge to self-indulge. Responses to the subscale help to distinguish between patients with BN and the dieting-indulging and purging group of patients with AN (bulimarexia), which later emerged as separate subtypes (restrictive and bulimic subtypes) according to DSM-IV criteria (American Psychiatric Association—APA, 1994) [19].
3. Body dissatisfaction: Items in this subscale reflect the belief that the shape of a body part (abdomen, hips, buttocks, thighs) should change or that it is too fat. Dissatisfaction with body image is often associated with other signs of body image disturbance (a core symptom of AN). Body dissatisfaction may be associated with low self-esteem and negative self-image.
4. Feeling of inadequacy: The items refer to a general feeling of inadequacy and worthlessness, inadequacy, lack of personal effectiveness. The feeling of inadequacy is a fundamental characteristic of eating disorders. It can be linked to the dimension of external control, because patients feel they have no control over what happens in their own lives. It is also associated with negative self-esteem.
5. Perfectionism: Items that show an excessive expectation of excellence and perfect performance fall into this sub-category. This is a defining characteristic of patients with AN, manifested in their over-adaptation to their parents’ high expectations and their strict system of supervision. Achievement orientation is typical of these families and patients. Perfectionism is an expression of dichotomous thinking: inertia and perfectionism are due to a cognitive distortion of “all or nothing” thinking and a primitive avoidance mechanism of splitting.
6. Interpersonal distrust: Subscale items express feelings of alienation and aversion to close relationships. Distrust is associated with relational inability and difficulties in expressing and sharing emotions with others.
7. Interoceptive awareness (lack of): Items indicating uncertainty in recognising and identifying internal sensations (hunger, satiety) and emotions are included in the subscale. This disorder is considered to be a fundamental feature of AN.
8. Items reveal a desire to retreat to the safety of pre-adolescence because the person finds the demands and expectations of adulthood overwhelming. The rejection of psychological maturity manifests itself in the rejection of food that causes development and growth.

#### 2.2.2. Túry and Colleagues’ Description of the Questionnaire

Each item is rated by the respondent by ticking one of six responses (‘always’, ‘usually’, ‘often’, ‘sometimes’, ‘rarely’, ‘never’) on a Likert scale of frequency (forced-choice self-report questionnaire). The most pathological answer (this is “always” in some items and “never” in the reverse-coded items) is worth 3 points, the answer next to it is worth 2 points, the next one is worth 1 point and the other three answers are worth 0 points. The score for each subscale is the sum of the values of the responses to the items to which it applies. Abnormal score limits may need to be set for the first three subscales, i.e., for the specific subscales for eating disorders. For the other subscales, where general personality factors are assessed, it is not necessary to draw a cut-off point. In general, the following thresholds are used: urge to be thin: 14 points; bulimia: 14 points; body dissatisfaction: 21 points. A score above the threshold in any of the first 3 subscales of the EDI indicates that the individual is likely to have an eating disorder [14].

#### 2.2.3. Suggestions on Concerns for Anorexia Nervosa (AN) According to the APA and Márta Varga

Another risk assessment scoring system of the EDI subscale was defined in the Hungarian context by Márta Varga’s dissertation in 2014, and in the American context by the American Psychiatric Association (APA) (2013) Diagnostic and Statistical Manual of Mental Disorders (DSM-V). Suggestions on concerns for AN if the BMI is less than 17.5 kg/m^2^ and the EDI bulimia factor is more than 14 points and the EDI the urge to be thin factor is more than 13 points, and/or the EDI body dissatisfaction factor is higher than 20 points [20,21].

#### 2.2.4. Suggestions on Concerns for Bulimia Nervosa (BN) According to the APA and Márta Varga

Another risk assessment scoring system of the EDI subscale was defined in the Hungarian context by Márta Varga’s dissertation in 2014, and in the American context by the American Psychiatric Association (APA) (2013) Diagnostic and Statistical Manual of Mental Disorders (DSM-V) [21]. Suggestions on concerns for BN are provided if the EDI bulimia factor is more than 13 points, the EDI urge to be thin factor is more than 13 points, and the EDI body dissatisfaction factor is higher than 20 points [17,18].

### 2.3. Other Items

In addition to the EDI questionnaire, participants’ current body weight and height were recorded. The Body Mass Index (BMI) of rowing athletes is self-reported—calculated as the ratio of their body weight in kilograms to their height in meters squared. BMI was expressed in kg/m^2^ as a scale variable. For further analysis, BMI values were grouped according to the current World Health Organization (WHO) BMI categories [22]. BMI may not be the most appropriate because of the potentially high muscle mass, but this is also a limitation of the research and has been used in many similar studies. Under 18 years, BMI alonentiles are considered. Basic sociodemographic data include gender, age, and when they started competitive rowing. It was also necessary to provide their performance on the ergometer this year and their most important result achieved during their racing career so far. The data collection also included questions about sleep habits and the level of knowledge and attitudes towards back and waist pain. Data on the following two topics will be published in other studies carried out jointly with my co-authors.

### 2.4. Statistical Analyses

MS Excel 2016 and IBM SPSS 26.0 software package were used to summarise the statistics and analyse the sample. Descriptive statistics included mean, standard deviation, and minimum and maximum values. Chi-square tests were used to test the hypothesis, and the Mann–Whitney U-test was used to test the difference between two weight groups. Pearson correlation was used for the relationships between variables. The level of statistical significance was set at *p* < 0.05 with a 95% confidence interval (CI: 95%).

## 3. Results

### 3.1. Sociodemographic Data

The survey recorded data and responses from a total of 222 (*n* = 222) rowing athletes. Sociodemographic data are shown in Table 1.

From the recorded data, we calculate the Body Mass Index (BMI) for each rower. The average BMI for both sexes was 22.31 kg/m^2^, which is considered normal body weight. The lowest extreme value was 15.53 kg/m^2^, which can be classified as severely underweight, and the highest calculated BMI was 32.93 kg/m^2^, which can be classified as obesity class I. When the average BMI data for the two sexes were compared, the expected result was obtained, with the average BMI for women being slightly lower than that for men (21.85 kg/m^2^; 22.70 kg/m^2^). No significant relationship was found between BMI data and gender, or between BMI data and weight category (*p* = 0.12; *p* = 0.32), but a significant relationship was found between BMI and age (*p* = 0.02; r = 0.891), so BMI is significantly higher at older ages.

In presenting a sample of rowing athletes, it is important to consider the category in which they compete, lightweight or openweight. Of the 222 (*n* = 222) athletes surveyed, 57 (*n* = 57) rowers competed in the lightweight category, i.e., almost a quarter (25.67%) of the athletes; a total of 165 (*n* = 165) athletes competed in the openweight category (74.33%). The lightweight rowers were almost evenly split between the sexes, with 28 (*n* = 28; 49.12%) women and 27 (*n* = 27; 50.88%) men. The gender distribution of competitors in the openweight category is as follows: 75 (*n* = 75; 45.45%) women and 90 men (*n* = 90; 54.55%).

**Figure 1:**
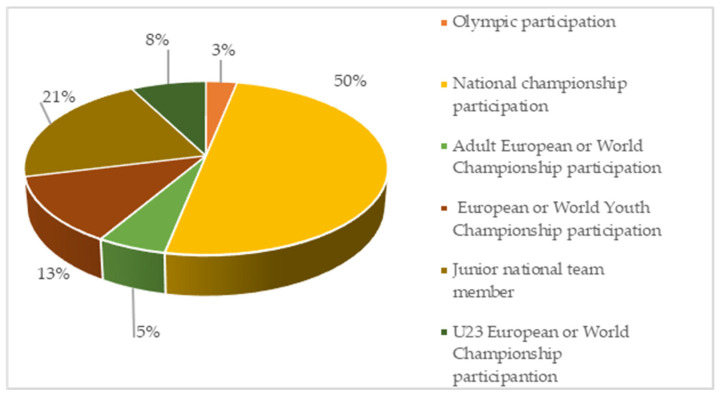
The most prestigious race by rowers (*n* = 222).

To further understand the sample, rowers were asked to indicate the most prestigious race by rowers in their sport life so far. They could choose from the following: I was an Olympic participant; I competed in a national championship; I was an adult European or World Championship participant; I was a European or World Youth Champion; I am a member of the junior national team; I was a U23 European or World Championship participant. Respondents could only select 1 option, which was considered their best sporting result (Figure 1).

Competitive sport and racing are already demanding at the national level, both physically and mentally, for athletes competing in any sport. Among the best achievements, many athletes have cited making it to national competitions, which is as mentally and psychologically taxing as it is physically. Athletes have very high expectations of themselves, their coaches and their families, which can have detrimental consequences under pressure, one of the consequences being the onset of eating disorders.

### 3.2. EDI Scale Results for the Two Weight Groups

The prevalence of eating disorders most commonly measured and observed in rowing athletes with anorexia nervosa (AN) and bulimia nervosa (BN) was investigated, based on previous research. Having coded the data of the validated EDI questionnaire according to the description, the mean scores and standard deviations of the eight subscales, as well as a comparison of the two weight groups, are shown in Table 2.

Subscales show a significant relationship between the two weight groups in two cases. On the perfectionism subscale and the interoceptive awareness subscale, rowers competing in the lightweight category are significantly more likely to score higher (*p* = 0.03; *p* = 0.05; r = 0.83; r = 0.76).

The core abnormal score ranges are required in the first three subscales specific to eating disorders; 14 points for the urge to be thin, 12 points for bulimia and 21 points for body dissatisfaction. A score above the threshold in any of the three subscales of the test indicates that the rowers are likely to have an eating disorder. Table 3 shows these three subscale points among the rowers and compares the two weight groups.

A score above the threshold in any of the first three subscales of the EDI test indicates that the patient may be at risk for an eating disorder.

The correlation between EDI score and BMI was examined, but no significant relationship was found between the two variables (*p* = 0.41). So, it can be said that those with a higher BMI score did not score higher on the EDI subscale. No significant relationship was found between EDI subscale scores and gender (*p* = 0.31). It was important to explore whether there is a correlation between the two weight groups and the score averages they achieve on the subscales of the EDI questionnaire. Are athletes in the lightweight group more affected by eating disorders? Did they score higher overall and separately on any of the subscales? Openweight competitors are more dissatisfied with their shape than lightweight competitors (*p* = 0.02; r = 0.89).

Comparing men and women, there is no significant difference between the sexes in how much less satisfied they are with their body shape, but women are more dissatisfied than men (*p* = 0.06). Comparing the subscales of the urge to be thin and gender, it was found that the urge to be thin subscale was significantly higher in women than in men; additionally, this subscale has no significant relationship between the two weight groups (*p* = 0.01; *p* = 0.60).

Comparing the bulimia subscale scores, no relationship was found with either weight group or gender, but men had a higher proportion of minimally higher total bulimia subscale scores (*p* = 0.60).

Comparing the body dissatisfaction subscale with weight groups and gender, no relationship was found, but women had a higher proportion of higher dissatisfaction subscale (*p* = 0.10; *p* = 0.61). The frequency of thoughts about dieting is not related to BMI, so athletes with a higher BMI do not think more about dieting (*p* = 0.55). However, a significant relationship was found between female athletes thinking more about diet than male athletes (*p* = 0.02; r = 0.76). The same indicator did not show a relationship with age and category (*p* = 0.85; *p* = 0.79). Athletes also had to answer a question about whether they turn to food as a stress reliever when they are anxious. No significant relationship was found between the two weight groups, with athletes in the openweight group not more likely to use eating as a stress reliever (*p* = 0.85). There is also no significant relationship between genders in this variable (*p* = 0.24).

Comparing the best results achieved by the rowers, i.e., whether they have competed in national championships, world championships or Olympics and whether they eat to reduce stress, which may be a sign of bulimia and binge eating, no significant relationship was found (*p* = 0.24), but rowers who have only competed in national championships and not in international competitions were more likely to reduce stress levels by eating. So, rowing athletes who are already practising the sport at a higher level may have more knowledge about stress relief and stress management methods, and so are less likely to resort to unnecessarily high levels and frequency of eating as a stress relief.

### 3.3. Prevalence of AN and BN among Competitve Rowers

Another risk assessment scoring system of the EDI subscale has already been presented, in the Hungarian context in a study by Márta Varga’a dissertation and the american context in the system of American Psychiatric Association (APA) (2013) Diagnostic and Statistical Manual of Mental Disorders (DSM-V) [17,18]. The results of the sample are shown in Table 4 and Table 5.

Several competitors reached the set scores, but there were no competitors who reached all four subscales. So, no competitor has met the AN criterion set by the APA requirements.

Among the athletes, there is no individual for whom all three criteria are true at the same time. There was one athlete who met two (body dissatisfaction factor = 27; the urge to be thin factor = 16 but bulimia factor = 6) of the three criteria described. She competes in an openweight category and has a BMI of 30.12 kg/m^2^, and she is 53 years old. The composite syndrome of bulimia nervosa is more typical of young adulthood, with a possible earlier onset in the teenage years, so this rower would not be considered at risk due to her older age.

It is important to add that without a clinical interview, the questionnaire survey alone is not sufficient to draw the final conclusion and classification.

## 4. Discussion

The aim of our study is to assess the rowing competitors certified by 21 sports clubs in Hungary for both sexes, to determine the prevalence of eating disorders—anorexia nervosa and bulimia nervosa. We also aimed to explore whether there is a relationship between athletes’ performance and the prevalence of eating disorders. Eating disorders among rowing athletes have not been studied in Hungary. However, as rowing is also a weight-related sport, especially in the lightweight category, monitoring of this is not negligible. Our survey took place in the summer of 2023 at the Hungarian national championships, with a total of 222 (*n* = 222) certified rowers participating. In this sample, the prevalence of eating disorders among rowers is not higher than in the general population, but given the limitations of the study, we recommend that further research is conducted to confirm these results. A limitation of the study is that the data collection took place in a stressful environment on race day. Potential confounding factors such as noise, inattention, and hurried, non-engrossed conversation should be mentioned. In 1996, Terry and Waite suggest that elite lightweight women rowers may be at increased risk for developing eating disorders [23]. This relationship was not confirmed in the present study, but openweight rowers are more dissatisfied with their shape than lightweight rowers. Comparing men and women, there is no significant difference between the sexes in how much less satisfied they are with their body shape, but women are more dissatisfied than men (*p* = 0.06). A 2024 study by Dal Brun and colleagues found that dissatisfaction with body weight significantly influences appearance judgement, eating behaviour and the psychopathology of muscle dysmorphia in men, which may also cause body image disturbances [24]. No significant association was found between weight groups, nor was the prevalence of eating disorders more pronounced in any of them. Scheffer et al. studied young female rowers in New Zealand in 2023 and wondered how low energy intake was a feature of New Zealand women athletes and whether energy intake was associated with brief eating disorders (Measures: brief eating disorder in athletes-questionnaire—BEDA-Q). The results of the study showed that low energy intake is common among elite female rowers in New Zealand (64%) and is positively correlated with higher BEDA-Q scores. The prevalence of eating disorders and low calorie intake was lower among Hungarian rowers; overall, 11 rowers (*n* = 11) out of 222 competitive athletes were identified as being at risk for eating disorders, 4.95% of the sample. In total, 9 of the 11 potentially affected athletes competed in the openweight class and a total of 2 competed in the lightweight class. Sundgot-Borgen in 2004 found a similarly low prevalence when she studied 522 professional female athletes [25]. Of the 522 Norway female athletes, 1.3% met criteria for anorexia nervosa and 8% met criteria for bulimia nervosa. In Sundgot-Borgen’s study 2004, the prevalence of bulimia was higher than in our sample [25]. Eating disorders should not be ignored as a risk factor because they can also have a strong impact on the performance of athletes, not only in training but also in competitions, where they have to perform under great psychological pressure to be at their best. In a research project, Alföldi et al. 2021 investigated how to select the rowing athlete with the best qualities, as well as what the characteristics are that determine who can be a more successful rower [26]. In addition to physical ability, the researchers also looked at psychological factors and found that mentally stronger competitors have an advantage in the field, while those who may be mentally ill-prepared, less able to cope with stress, less aware of stress management techniques and coping strategies are at a disadvantage in the race. In this study, we also investigated whether rowing athletes use nutrition to reduce stress levels, and, if so, whether there is a difference between weight groups [27,28]. The same question was asked by Walsh et al. 2020, but with similar results that there was no significant difference between the two weight groups [27]. In this study, it was observed that in the openweight group, the combination of these three factors among the three criteria defined by the APA put the athlete at risk for bulimia, with the prevalence of the criteria separately being higher in the openweight category.

## 5. Conclusions

Eating disorders in competitive athletes are an important clinical problem for sports physicians, coaches, trainers and dietitians, all of whom have an interest in protecting athletes from the potential health consequences of participation in sport. This study found no evidence that rowing athletes are markedly more affected by the prevalence of eating disorders, but further studies are needed to establish the exact prevalence, as the prevention of eating disorders and the limitation of abnormal weight-loss behaviors are ongoing clinical problems. Ongoing and appropriate education of athletes, including regular nutritional counselling, can help to ensure that athletes are aware of healthy ways to manage their weight.

## Figures and Tables

**Table 1:** Sociodemographic characteristics (*n* = 222).

Sex	Sample (n)	Percentage (%)
Women	104	46.85%
Men	118	53.15%
Age	Years	Standard Deviation
Youngest	13	
Oldest	67	
Average	23.29	12.23
Weight	Kg	Standard Deviation
Lowest	34	
Highest	115	
Average	70.25	13.82
Height	Cm	Standard Deviation
Lowest	148	
Highest	203	
Average	176.87	9.39

**Table 2:** Scores on the EDI 8 subscales and their SD among the rowers surveyed (*n* = 222) and comparing the two weight groups. * Significant at 0.03; ** Significant at 0.05.

	Available Score Range of Subscale	Score Average of the Subscale (*n* = 222) (SD±)	Score Average of the Subscale among Lightweight Rowers (*n* = 57) (SD±)	Score Average of the Subscale among Openweight Rowers (*n* = 165) (SD±)	*p*-Value
1. The urge to be thin subscale	0–18	2.55 (3.46)	2.59 (3.47)	2.53 (3.46)	0.56
2. Bulimia subscale	0–21	1.83 (3.38)	1.61 (3.40)	1.91 (3.37)	0.71
3. Body dissatisfaction subscale	0–27	4.41 (5.21)	3.82 (5.22)	4.62 (5.20)	0.49
4. Feeling of inadequacy subscale	0–30	2.86 (4.35)	3.56 (4.36)	2.62 (4.34)	0.11
5. Perfectionism subscale	0–18	3.29 (4.17)	3.91 (4.18)	3.08 (4.16)	0.03 *
6. Interpersonal distrust subscale	0–21	3.16 (3.79)	3.50 (3.77)	3.04 (3.78)	0.64
7. Interoceptive awareness (lack of) subscale	0–30	3.04 (5.05)	3.82 (5.08)	2.76 (5.04)	0.05 **
8. Fear of adulthood subscale	0–21	7.01 (4.72)	7.68 (4.72)	6.78 (4.70)	0.24

**Table 3:** The abnormal score ranges in the first three subscales.

	In Both Weight Category (*n* = 222) (n)	Among Lightweight Rowers (*n* = 57) (n)	Among Openweight Rowers (*n* = 165) (n)
1. The urge to be thin subscale ≥14 points	3	1	2
2. Bulimia subscale ≥14 points	4	0	4
3. Body dissatisfaction subscale ≥21 points	4	1	3

**Table 4:** Suggestions on concerns for anorexia nervosa (AN) according to the APA and Márta Varga.

Suggestions on Concerns of AN	Lightweight Category (*n* = 57) (n)	Openweight Category (*n* = 165) (n)	Both Weight Category (*n* = 222) (n)
BMI < 17.5 kg/m^2^	6	0	6
Bulimia factor < 14	57	161	218
The urge to be thin factor > 13	1	4	5
Body dissatisfaction factor > 20	0	2	2

**Table 5:** Suggestions on concerns for bulimia nervosa (BN) according to the APA and Márta Varga.

Suggestions on Concerns of BN	Lightweight Category (*n* = 57) (n)	Openweight Category (*n* = 165) (n)	Both Weight Category (*n* = 222) (n)
Bulimia factor > 13	0	4	4
The urge to be thin factor > 13	1	4	5
Body dissatisfaction factor > 20	0	2	2

## Data Availability

The raw data supporting the conclusions of this article will be made available by the authors upon request.

## References

[1] American Psychiatric Association (2023). What Are Eating Disorders?. https://www.psychiatry.org/patients-families/eating-disorders/what-are-eating-disorders.

[2] Túry F., Lukács L., Horváth K., Rácz O. (2003). Az evés- és a testképzavarok újabb megnyilvánulásai. Lege Artis. Med..

[3] Varga M., Dukay-Szabó S., Túry F. (2013). Orthorexia nervosa és háttértényezői. Ideggyógyászati Szle./Clin. Neurosci..

[4] Ali A.M., Alameri R.A., Brooks T., Ali T.S., Ibrahim N., Khatatbeh H., Pakai A., Alkhamees A.A., Al-Dossary S.A. (2023). Cut-off scores of the Depression Anxiety Stress Scale-8: Implications for improving the management of chronic pain. J. Clin. Nurs..

[5] Karlson K.A., Becker C.B., Merkur A. (2001). Prevalence of eating disordered behavior in collegiate lightweight women rowers and distance runners. Clin. J. Sport. Med..

[6] Slater G., Rice A., Jenkins D., Hahn A. (2014). Body mass management of lightweight rowers: Nutritional strategies and performance implications. Br. J. Sports Med..

[7] Rucinski A. (1989). Relationship of body image and dietary intake of competitive ice skaters. J. Am. Diet. Assoc..

[8] Tan J.O., Calitri R., Bloodworth A., McNamee M.J. (2016). Understanding Eating Disorders in Elite Gymnastics: Ethical and Conceptual Challenges. Clin. Sports Med..

[9] Ohashi Y.B., Wang S.B., Shingleton R.M., Nock M.K. (2023). Body dissatisfaction, ideals, and identity in the development of disordered eating among adolescent ballet dancers. Int. J. Eat. Disord..

[10] Podstawski R., Borysławski K., Katona Z.B., Alföldi Z., Boraczyński M., Jaszczur-Nowicki J., Gronek P. (2022). Sex Differences in Anthropometric and Physiological Profiles of Hungarian Rowers of Different Ages. Int. J. Environ. Res. Public Health.

[11] Byrne S., McLean N. (2002). Elite athletes: Effects of the pressure to be thin. J. Sci. Med. Sport.

[12] Wells K.R., Jeacocke N.A., Appaneal R., Smith H.D., Vlahovich N., Burke L.M., Hughes D. (2020). The Australian Institute of Sport (AIS) and National Eating Disorders Collaboration (NEDC) position statement on disordered eating in high performance sport. Br. J. Sports Med..

[13] Joy E., Kussman A., Nattiv A. (2016). 2016 update on eating disorders in athletes: A comprehensive narrative review with a focus on clinical assessment and management. Br. J. Sports Med..

[14] Sundgot-Borgen J., Torstveit M.K. (2004). Prevalence of eating disorders in elite athletes is higher than in the general population. Clin. J. Sport Med..

[15] Fritz P., Kiss A., Pfeiffer L. (2020). A sportolók körében előforduló evészavarok = Eating disorders in athletes. Recreation.

[16] Clemente-Suárez V.J., Ramírez-Goerke M.I., Redondo-Flórez L., Beltrán-Velasco A.I., Martín-Rodríguez A., Ramos-Campo D.J., Navarro-Jiménez E., Yáñez-Sepúlveda R., Tornero-Aguilera J.F. (2023). The Impact of Anorexia Nervosa and the Basis for Non-Pharmacological Interventions. Nutrients.

[17] Garner D.M., Olmstead M.P., Polivy J. (1983). Development and validation of a multidimensional eating disorder inventory for anorexia nervosa and bulimia. Int. J. Eat. Disord..

[18] Túry F., Sáfrán Z., Wildmann M., László Z. (1997). Az Evési Zavar Kérdőív (Eating Disorder Inventory) hazai adaptációja. Szenvedélybetegségek.

[19] Bell C.C. (1994). DSM-IV: Diagnostic and Statistical Manual of Mental Disorders. JAMA.

[20] Varga M. (2014). Az Orthorexia Nervosa Korrelátumai, Különös Tekintettel az Evészavarokra és a Kényszeres Tünetekre. Ph.D. Dissertation.

[21] American Psychiatric Association (2013). Diagnostic and Statistical Manual of Mental Disorders.

[22] Weir C.B., Jan A. (2023). BMI Classification Percentile and Cut off Points. StatPearls.

[23] Terry P.C., Waite J. (1996). Eating attitudes and body shape perceptions among elite rowers: Effects of age, gender and weight category. Aust. J. Sci. Med. Sport.

[24] Dal Brun D., Pescarini E., Calonaci S., Bonello E., Meneguzzo P. (2024). Body evaluation in men: The role of body weight dissatisfaction in appearance evaluation, eating, and muscle dysmorphia psychopathology. J. Eat. Disord..

[25] Sundgot-Borgen J. (1994). Risk and trigger factors for the development of eating disorders in female elite athletes. Med. Sci. Sports Exerc..

[26] Alföldi Z., Borysławski K., Ihasz F., Soós I., Podstawski R. (2021). Differences in the Anthropometric and Physiological Profiles of Hungarian Male Rowers of Various Age Categories, Rankings and Career Lengths: Selection Problems. Front. Physiol..

[27] Walsh M., Crowell N., Merenstein D. (2020). Exploring Health Demographics of Female Collegiate Rowers. J. Athl. Train.

[28] Castañeda-Babarro A., León-Guereño P., Viribay A., Gutiérrez-Santamaría B., López I., Mielgo-Ayuso J. (2024). The Influence of Anthropometric Variables on the Performance of Elite Traditional Rowers. Sports.

